# Highly Selective Cyclooxygenase-1 Inhibitors P6 and Mofezolac Counteract Inflammatory State both *In Vitro* and *In Vivo* Models of Neuroinflammation

**DOI:** 10.3389/fneur.2017.00251

**Published:** 2017-06-09

**Authors:** Rosa Calvello, Dario Domenico Lofrumento, Maria Grazia Perrone, Antonia Cianciulli, Rosaria Salvatore, Paola Vitale, Francesco De Nuccio, Laura Giannotti, Giuseppe Nicolardi, Maria Antonietta Panaro, Antonio Scilimati

**Affiliations:** ^1^Department of Biosciences, Biotechnologies and Biopharmaceutics, University of Bari “A. Moro”, Bari, Italy; ^2^Department of Biological and Environmental Sciences and Technologies, Section of Human Anatomy, University of Salento, Lecce, Italy; ^3^Department of Pharmacy – Pharmaceutical Sciences, University of Bari “A. Moro”, Bari, Italy

**Keywords:** cyclooxygenase-1, P6 and mofezolac, cyclooxygenase-1 inhibitors, neuroinflammation, *in vitro* and *in vivo* experiments, lipopolysaccharide-treated BV2 microglial cells

## Abstract

Activated microglia secrete an array of pro-inflammatory factors, such as prostaglandins, whose accumulation contributes to neuronal damages. Prostaglandin endoperoxide synthases or cyclooxygenases (COX-1 and COX-2), which play a critical role in the inflammation, are the pharmacological targets of non-steroidal anti-inflammatory drugs, used to treat pain and inflammation. Since it was reported that COX-1 is the major player in mediating the brain inflammatory response, the aim of this study was to evaluate the effects of highly selective COX-1 inhibitors, such as P6 and mofezolac, in neuroinflammation models. Lipopolysaccharide (LPS)-activated mouse BV-2 microglial cells and LPS intracerebroventricular-injected mice as *in vitro* and *in vivo* neuroinflammation models, respectively, were used to probe the antiinflammatory efficacy of P6 and mofezolac. Both P6 and mofezolac reduce COX-1 expression in LPS-activated BV-2 cells. This reduction was accompanied with PGE_2_ release reduction and NF-kB activation downregulation. Coextensively, in the *in vivo* model, both glial fibrillary acidic protein and ionized calcium-binding adapter molecule-1 expression, two markers of inflammation, were reduced by mofezolac to a rank depending on the encephalon area analyzed. The increase of COX-1 expression observed in all the brain sections of LPS-treated mice was selectively downregulated by the *in vivo* treatment with mofezolac as well as PGE_2_ release and Ikβα phosphorylation amount assayed in the brain areas tested. These results indicate the capability of P6 and mofezolac to modulate the NF-kB signaling pathway, emphasizing the neuroprotective effect and therapeutic potential of COX-1 inhibitors in the control of neuroinflammatory diseases.

## Introduction

Neuroinflammation is widely recognized as an inflammatory response originated in the central nervous system (CNS). It is a pathological condition mainly caused by the nervous tissue infiltration of host defense cells and molecules from the bloodstream. In addition, it implies a complex interplay of glia, in particular microglia, typically associated with neurological and neurodegenerative diseases, triggering several concerns from a nosological viewpoint (1).

Microglia are recognized as the innate immune cells of the CNS, where they mediate a number of tissue homeostatic functions, including immune surveillance (2), synaptic regulation (3, 4), and neurogenesis (5, 6). Microglial activation and chronic inflammation thereafter is the starting point for elevated levels of a wide array of potentially neurotoxic molecules including pro-inflammatory cytokines, proteinases, and reactive oxygen species (ROS), which are believed to contribute to neurodegenerative processes (7–9).

Epidemiological data-based link between neuroinflammation and neurodegenerative diseases increased the worldwide scientific interest aimed to determine whether reducing inflammation would reverse neurodegeneration. Such data also indicate an inverse relationship between the use of traditional non-steroidal anti-inflammatory drugs (*t*NSAIDs) and Alzheimer’s disease risk. *t*NSAIDs pharmacological action is due to their ability to inhibit the cyclooxygenase (COX) and, hence, the biosynthesis of the prostaglandins (PG) involved in neuroinflammation (10).

Two COXs are known, COX-1 and COX-2. Upon inflammatory stimuli, COX-1, being constitutive in microglia, is responsible of the primary inflammatory response by inducing the production of PG, mainly PGE_2_. COX-2 is responsible, upon its induction, of a later and secondary response, with the exception of conditions in which the neurons are directly challenged (excitotoxicity and ischemia) (11). In the latter case, the primary response is COX-2 mediated, and microglia derived COX-1 succeeds as a secondary response upon perpetuating stimuli. In the first circumstances, selective COX-1 catalytic activity inhibition may result beneficial. *t*NSAIDs inhibit COXs not selectively. Then, drugs useful to treat the different neuroinflammatory conditions are needed (12, 13).

Preliminarily, we accomplished an *in vitro* study using some selective COX-1 inhibitors (P6, P10, and SC-560) compared to aspirin and two selective COX-2 inhibitors (celecoxib and etoricoxib) in a *in vitro* model of neuroinflammation, represented by lipopolysaccharide (LPS)-stimulated N13 microglial cell line (14). In such a context, the treatment of LPS-stimulated microglial cells by selective COX-1 inhibitors P6 and P10 were able to downregulate COX-1 protein expression without affecting COX-2 levels; interfering with NF-kB activation (14).

In continuation of our investigations, herein, we report the validation of the study above mentioned by using both an *in vitro* model of neuroinflammation, represented by LPS-activated BV2 cell line, and an *in vivo* animal model, constituted by LPS-treated mice.

**Figure 1:**
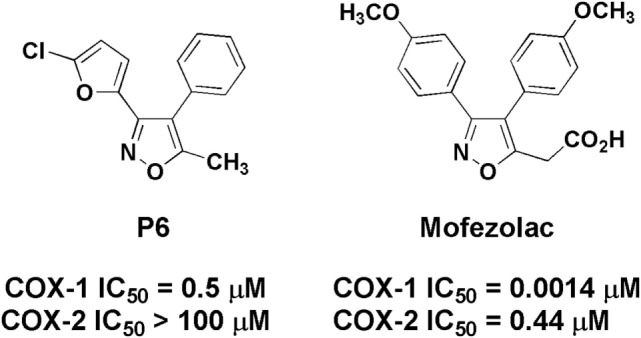
Chemical structure of P6 and mofezolac. IC_50_ values refer to the human whole blood assay.

In particular, in this study, COX-1 role in neuroinflammation was explored by using P6 and mofezolac (Figure 1). Both compounds belong to the diarylisoxazole class, and mofezolac is the only clinically used diarylisoxazole approved in Japan as Disopain^®^.

## Materials and Methods

### Reagents

P6 was synthesized according to Di Nunno et al. (15), whereas mofezolac was synthesized following Micetich’s protocol (16). All the other reagents and solvents were purchased from Sigma-Aldrich (Milan, Italy) and used without any further purification.

Lipopolysaccharide from *Escherichia coli* serotype 0127:B8 was purchased from Sigma-Aldrich (Milan, Italy). The goat polyclonal a p-IκB (sc-7977) antibody (Ab) was purchased from Santa Cruz Biotechnology (DBA, Milan, Italy); COX-1 (Ab 695) and COX-2 (Ab 15191) Abs were obtained from Abcam (Cambridge, UK). Goat anti-rabbit IgG (sc-2004), goat anti-mouse IgG (sc-2005), and donkey anti-goat IgG (sc-2020) were purchased from Santa Cruz Biotechnology; mouse primary monoclonal antibody (mAb) anti-glial fibrillary acidic protein (GFAP) (Merck Millipore, Milan, Italy), mouse mAb anti-ionized calcium-binding adapter molecule-1 (Iba-1) (Merck Millipore). Elisa kit for PGE_2_ evaluation was purchased from Cayman Chemical (Ann Arbor, MI, USA). MTT [3-(4,5-dimethylthiazol-2-yl)-2,5-diphenyltetrazolium bromide], 3,3′-diaminobenzidine, and tribromoethanol were obtained from Sigma-Aldrich, Milan, Italy.

### Cell Cultures and Treatment

BV2 microglia cells (ICLC HTL 03001-Interlab Cell Line Collection) were grown in high glucose Dulbecco’s modified Eagle’s medium supplemented with 10% fetal bovine serum, 100 U/mL penicillin, and 100 µg/mL streptomycin. They were maintained at 37°C in a humidified 5% CO_2_/95% environmental air.

Then, microglial cells were plated, at a density of 25 × 10^4^/well in 6-well plates (Falcon) and treated with the chosen COX inhibitors (P6 and mofezolac) when they reached 80% confluence. Preliminary experiments were conducted to establish the optimal concentration and exposure times necessary for LPS (1 µg/mL) treatment, which were found to be in accordance with other reports (17, 18), as well as to establish the optimal dose of the COX inhibitors and exposure times to detect their effects on LPS-stimulated BV2 microglial cell function. In this regard, a set of experiments was carried out in which microglial cells were 1 h pretreated with the selective COX-1 inhibitors P6 (0.5 and 1.0 µM) or mofezolac (0.1 and 0.5 µM). Cells were then incubated for different times at 37°C with LPS, as pro-inflammatory stimulus. Experiments included cells grown in medium alone (control).

### Cell Viability Test

Cell viability of microglial cells was quantified using the MTT reduction assay. The cells (8 × 10^3^/well) were grown in 96-well plates (Becton Dickinson Labware) in complete medium and treated with different concentration of COX inhibitors, in presence or absence of LPS. Untreated cells were used as a control. A PBS 1× solution of MTT (5 mg/mL) was prepared and added to the cell medium at a final concentration of 0.5 mg/mL. Cells were incubated for 4 h at 37°C and 5% CO_2_ to allow the MTT metabolism. The formazan crystals formed (from MTT) into the cells were solubilized with DMSO (Sigma–Aldrich). The levels of MTT formazan were determined measuring the optical density at λ = 560 nm and subtracting the background (λ = 670 nm) with a Victor Multiplate Reader (Wallac). Optical density was directly correlated to cell quantity.

### Immunoblotting Assay

After treatment of cultures as previously described, cells were harvested and lysed by ice-cold lysis buffer [1% Triton X-100, 20 mM Tris–HCl, 137 mM NaCl, 10% glycerol, 2 mM EDTA, 1 mM phenylmethylsulfonyl fluoride, 20 µM leupeptin hemisulfate salt, and 0.2 U/mL aprotinin (all from Sigma–Aldrich)] for 30 min on an ice bath.

Substantia nigra pars compacta, hippocampus, frontal lobe, and caudate from mice brains were minced in ice-cold PBS, washed, and then homogenized in a buffer containing lysis buffer (50 mM Tris pH 8, 0.02 g/mL NaCl, 0.2% SDS, 1% Triton-X, 4 U/mL aprotinin, 2 mM leupeptin, and 100 mM phenylmethanesulfonyl fluoride).

The tissue and cell culture lysates were vortexed for 15–20 s and, then, centrifuged at 12,800 × *g* for 20 min. The protein concentration in the supernatant was spectrophotometrically determined by Bradford’s protein assay. Protein samples were diluted with sample buffer (0.5 M Tris–HCl pH = 6.8, 10% glycerol, 10% (w/v) SDS, 5% 2-mercaptoethanol, and 0.05% (w/v) bromophenol blue) and then boiled for 3 min. Proteins (25 µg/lane) and prestained standards (BioRad Laboratories, Hercules, CA, USA) were loaded on 7% or 12% SDS precast polyacrylamide gels (BioRad Laboratories).

After electrophoresis, the resolved proteins were transferred from the gel to nitrocellulose membranes. A blotting buffer [20 mM Tris/150 mM glycine, pH = 8.0, and 20% (v/v) methanol] was used for gel and membrane saturation and blotting. Then, membranes were incubated in the dark with the following specific primary Abs reported in reagents section for 60 min at room temperature. The membranes were washed with 0.1% Tween 20-PBS (for 20 min, three times) and then incubated with the secondary Ab diluted 1:2,000 for 60 min. Bands were visualized by chemiluminescence detection (Invitrogen, Milan, Italy). The β-actin level was used as a protein loading control. For tissue analysis, obtained bands were normalized to the level of β-actin performed for each cerebral area tested. For cell cultures, the bands were normalized to the β-actin level of each experimental condition. The bands obtained after immunoblotting were submitted to densitometric analysis using 1D Image Analysis Software (Kodak Digital Science). Results were expressed as arbitrary units.

### Animals and Treatment Protocols

This study was carried out in strict accordance with the European Council Directive 86/609/EEC and the Italian animal welfare legislation (art. 4 and 5 of D.L. 116/92). Seventy adult male 129S2/Sv mice (22–24 g body mass, 8–10 weeks of age) were purchased from Harlan—Italy and were kept under environmentally controlled conditions (20 ± 2°C, 50–80% humidity, 12 h light/dark cycle, food and water *ad libitum*) and divided into 7 groups of 10 animals (5 for western blotting and 5 for immunohistochemical procedures).

Mice received either the selective COX-1 inhibitor mofezolac (6 mg/kg, i.p.; indicated as M in all the figures) or vehicle (40% DMSO in 0.1 M phosphate buffer, pH = 7.4; VM in all the figures) once a day for 10 days. Mofezolac amount to be injected was chosen taken into account previous study and its COXs IC_50_ values. On the seventh day, mice were anesthetized with tribromoethanol (250 mg/kg, i.p.) and positioned in a stereotactic apparatus (Kopf Instruments, Tujunga, CA, USA). Vehicle (sterile saline, 5 µL; V-LPS in all the figures) or 5 µg LPS in 5 µL of sterile saline (LPS in all the figures) was administered into the cerebral lateral ventricle using a fine needle glass syringe (Hamilton, Lyon, France) and a syringe pump (KD Scientific, Holliston, MA, USA) at a rate of 1 µL/min. This LPS dose and time point (72 h) induced the best neuroinflammatory response following a titration (24 up to 60 h) preliminary study (data not shown). Stereotaxic injections coordinates were 2.3 mm dorsal/ventral, 1.0 mm lateral, and 0.5 mm anterior/posterior from the bregma. Mofezolac was given 30 min prior to LPS injection (M + LPS in all the figures).

### Immunohistochemical Staining

Mice were transcardially perfused with tris-buffered saline (pH = 7.6) followed by 4% paraformaldehyde in PBS pH = 7.4 at 4°C. Brains were subsequently postfixed in the same fixative, paraffin embedded, and 10 µm slices were obtained with a rotative microtome (Leica, Milan, Italy). Immunohistochemistry was performed following a standard avidin–biotin complex procedure. Briefly, specimens were incubated with mouse primary mAb anti-GFAP at a ratio of 1:1,000 (Merck Millipore, Milan, Italy), or a mouse mAb anti-Iba-1 at a ratio of 1:500 (Merck Millipore) overnight at 4°C and then with an anti-mouse biotinylated secondary Ab (Dako, Milan, Italy), at a 1:1,000 dilution for 1 h at room temperature. The antigen–Ab complexes were visualized by sections incubation for 1 h with extravidin peroxidase (Sigma-Aldrich) diluted 1:1,500 and 3,3′-diaminobenzidine oxidation in the presence of H_2_O_2_.

### PGE_2_ Assay

Microglial cells were cultured in 6-well plates at a density of 3 × 10^6^ cells/well. Then, the cells were pretreated with selective COX-1 inhibitors P6 or mofezolac for 1 h and, subsequently stimulated with LPS (1 µg/mL). The cultures were maintained at 37°C for 24 and 48 h in a humidified air containing a 5% CO_2_. PGE_2_ levels were determined in the supernatant using a competitive binding immunoassay (Cayman Chemical, Ann Arbor, MI, USA) following the manufacturer’s instructions. Unstimulated cells were included as a control. PGE_2_ amount determination in the brain was performed in the tissue extracts, according to the manufacturer’s instructions. The optical density was measured at λ = 405–420 nm with precision microplate reader and the amount of PGE_2_ (ng/mL) was calculated using a PGE_2_ standard curve.

### Statistical Analysis

Student’s *t*-test and analysis of variance (one-way ANOVA) on the results of at least five independent biological replicates were performed. Values of *p* < 0.05 were considered statistically significant.

## Results

### BV2 Microglial Cell Viability Assay

MTT assay was used to quantitatively evaluate cell viability. This was performed to verify whether the tested selective COX-1 inhibitors (P6 and mofezolac) caused toxicity in LPS-treated BV2 cell line. Preliminarily, the effect of two different concentrations of P6 (0.5 and 1 µM) and mofezolac (0.1 and 0.5 µM) on BV2 microglial cell viability was evaluated. No cell toxicity was exerted by either P6, mofezolac, and LPS alone or a combination of LPS and each of the two inhibitors at 24 h. The two concentrations of P6 and mofezolac were chosen based on the basis of previous studies and their COXs IC_50_ values (14).

Cell viability was found to be significantly (*p* ≤ 0.001) lower, after 48 h of LPS treatment (percentage cell viability equal to 78.4 ± 0.15 vs 99.8 ± 0.07 of the control), than untreated cells (control). None of the inhibitors used at the above indicated doses were toxic to BV2 microglial cells, when used in the absence of LPS (percentage cell viability ranging between 95.5 ± 0.22 and 98.2 ± 0.16 vs control). In addition, inhibitors used at the same doses, resulted protective toward BV2 microglial cells after 48 h LPS treatment (1 µg/mL), being the percentage of cell viability comparable to that observed in control.

### Evaluation of Mice Glial Activation

**Figure 2:**
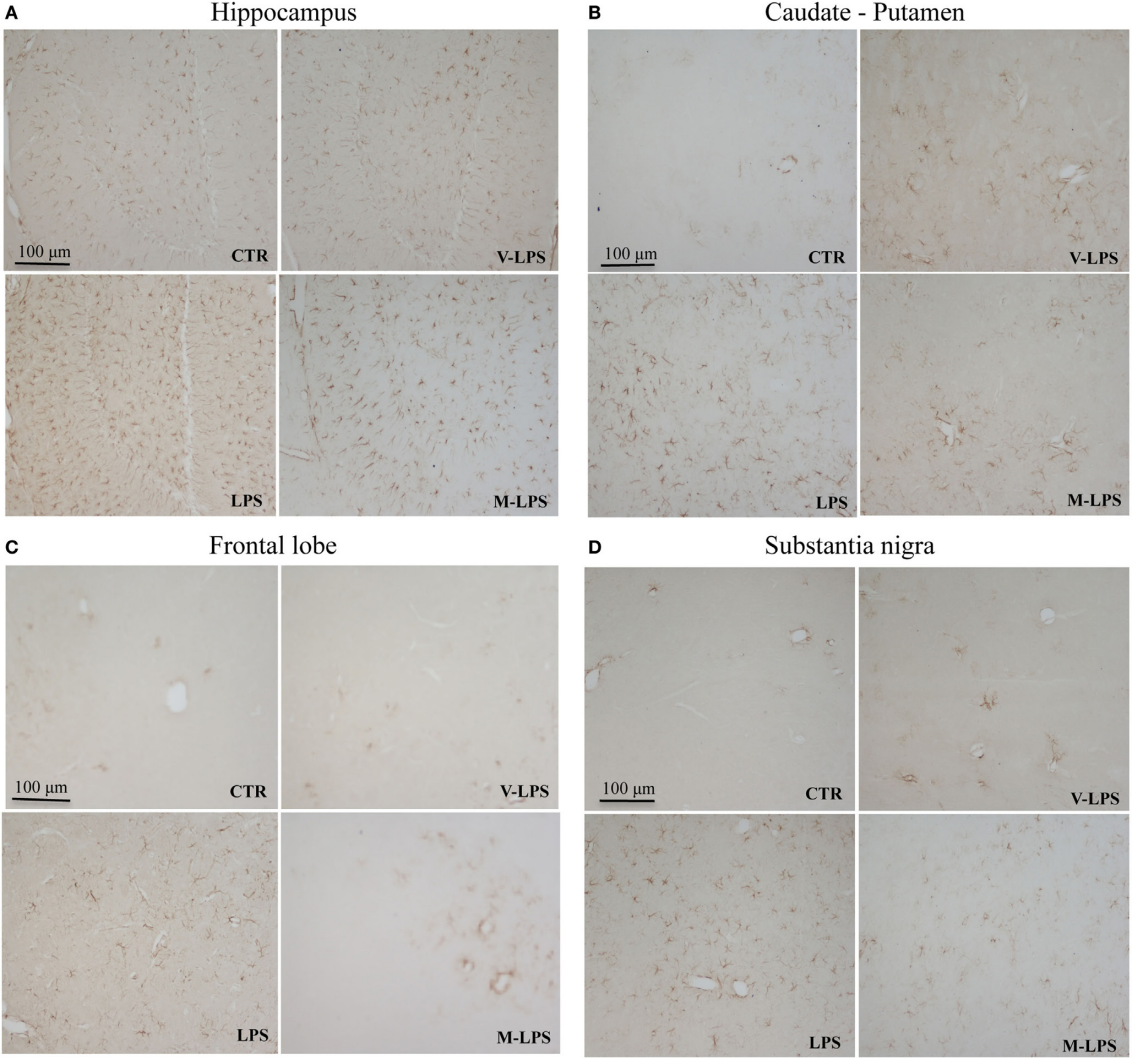
Glial fibrillary acidic protein immunoreactivity in the hippocampus **(A)**, caudate–putamen **(B)**, frontal lobe **(C)**, and substantia nigra **(D)**, in slices of control (CTR), vehicle of LPS (V-LPS), lipopolysaccharide (LPS), mofezolac and LPS (M-LPS)-treated mice.

Astroglial activation was characterized by immunoreactivity and immunoblotting analysis of the GFAP expression, a marker used to distinguish astrocytes from other glial cells of the CNS. LPS treatment determined an increase of immunoreactive cell bodies in comparison to untreated mice suggesting astrocyte activation in different brain regions. In particular, the caudate, frontal lobe, hippocampus, and substantia nigra were selected to evaluate the astrocyte activation after different animal treatment (Figures 2A–D). Images report GFAP reactivity in samples of mice control group (CTR), mice treated with LPS vehicle (V-LPS), LPS alone (LPS) or in combination with mofezolac (M + LPS). In CTR sections, there is a physiological astroglial distribution with few immunoreactive elements surrounding the blood vessels as part of the brain–blood barrier, which means that there are no sign of reactive astrogliosis. In V-LPS-treated mice, the expression is similar to CTR, with perivascular immunoreactivity and the presence of a few astrocytes in the tissue, due to inflammation probably caused from the injection. In LPS-treated mice, images show a marked increase of the immunoreactive elements, whereas *in vivo* administration of mofezolac reduced the presence of GFAP immunoreactive cells in all the tested brain areas (Figures 2A–D).

**Figure 3:**
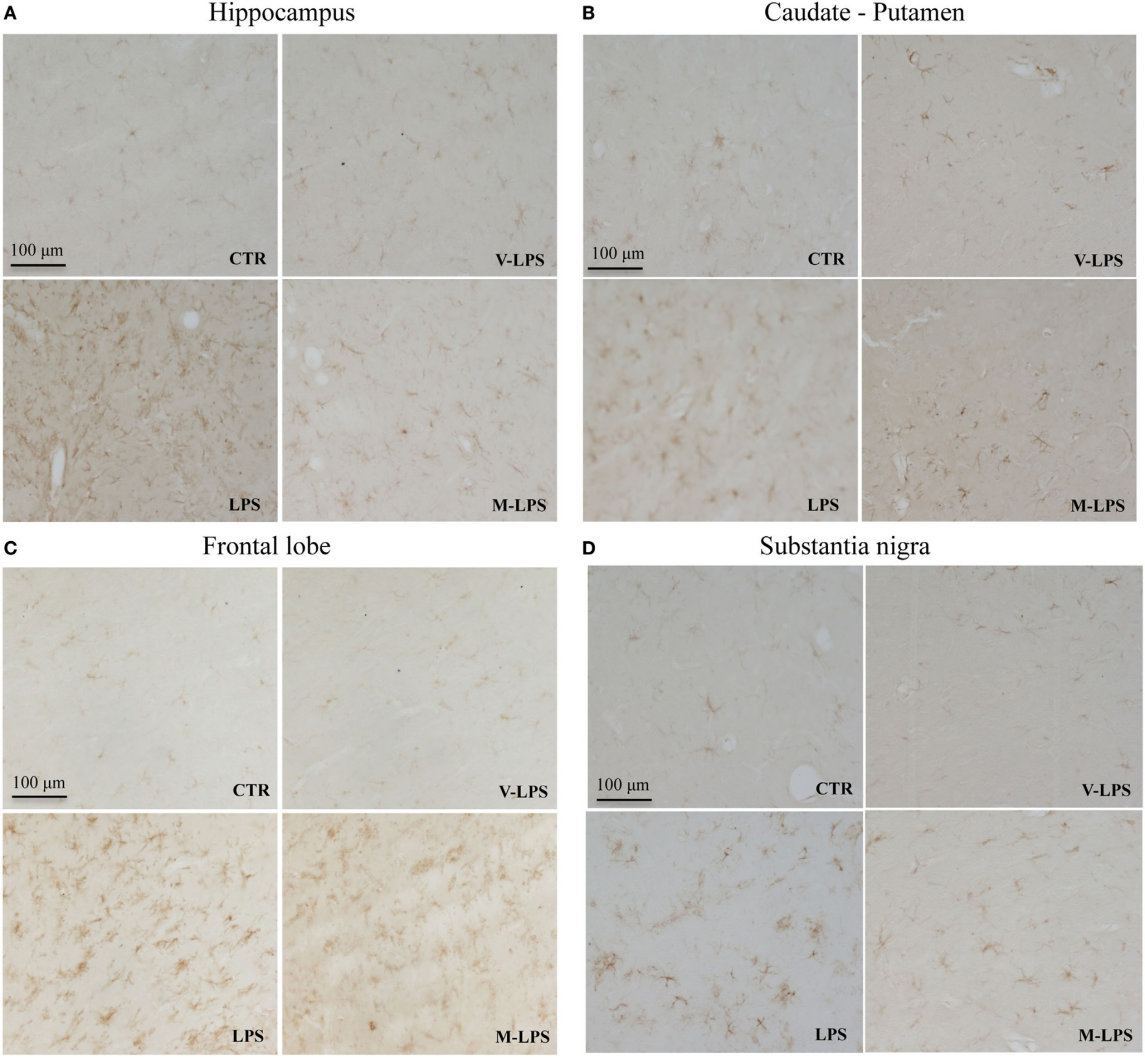
Ionized calcium-binding adapter molecule-1 immunoreactivity in the hippocampus **(A)**, caudate–putamen **(B)**, frontal lobe **(C)**, and substantia nigra **(D)**, in slices of control (CTR), vehicle of LPS (V-LPS), lipopolysaccharide (LPS), and mofezolac and LPS (M-LPS)-treated mice.

Ionized calcium-binding adapter molecule-1 immunoreactivity, a marker of activated microglia, was also evaluated (Figure 3). LPS-treated mice Iba-1 positive cells were more numerous, showing a more intense immunoreactivity, as well as a ramified phenotype, in comparison to untreated mice (Figures 3A–D). Mofezolac administration determined the reduction of Iba-1 immunoreactivity in LPS-injected mice in all the tested brain areas (Figures 3A–D), suggesting that mofezolac reduces, at least in part, the microglial activation induced by the neurotoxic insult.

**Figure 4:**
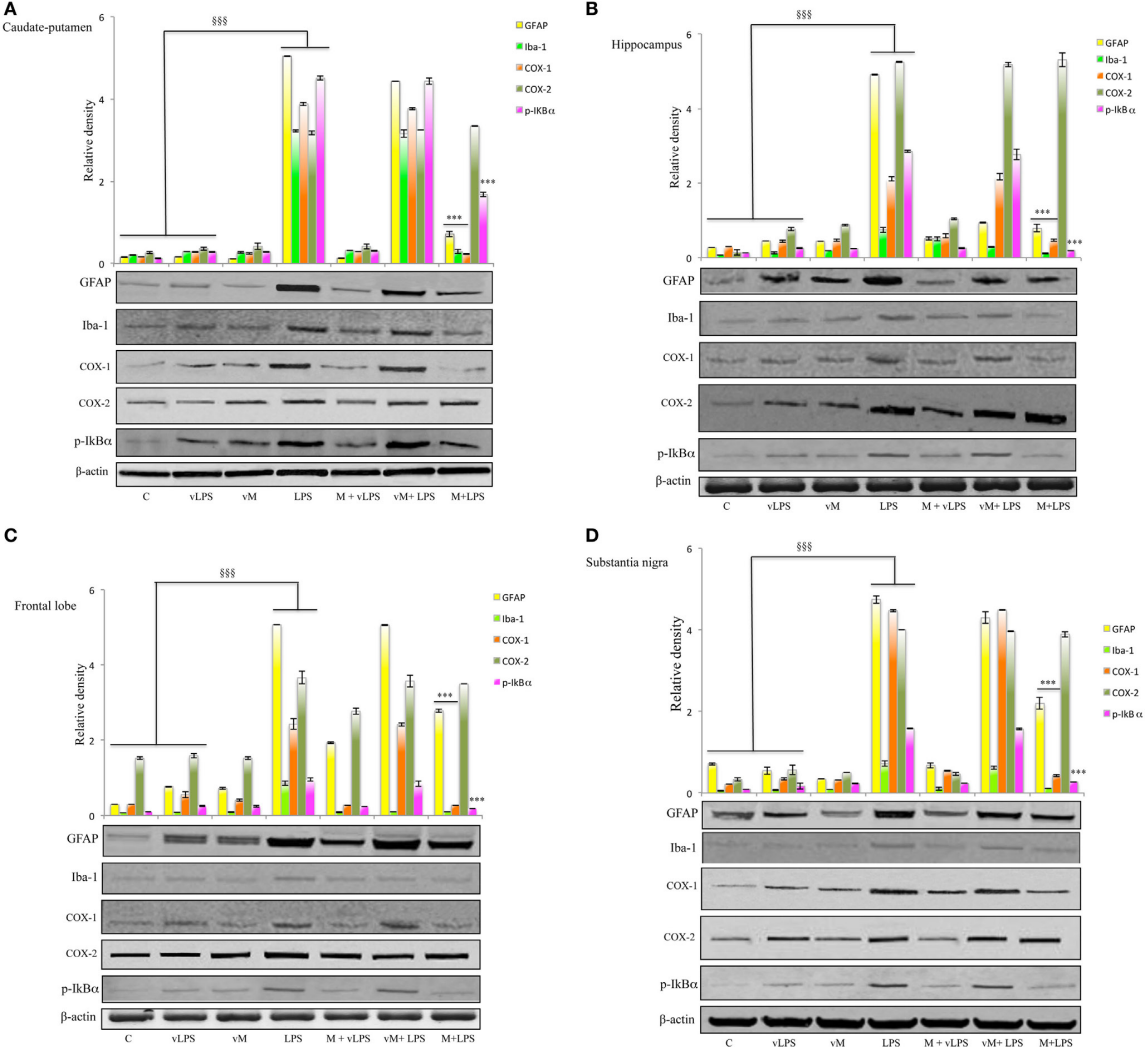
Effect of mofezolac on glial fibrillary acidic protein (GFAP), ionized calcium-binding adapter molecule-1 (Iba-1), cyclooxygenases (COXs), and pI*k*Bα expression in **(A)** caudate–putamen, **(B)** hippocampus, **(C)** frontal lobe, and **(D)** substantia nigra from mice treated with lipopolysaccharide (LPS) alone and LPS in the presence of mofezolac. The quantification of relative band intensities was expressed as relative density, after normalization against β-actin densitometry. Values represents the means ± SE of three independent experiments. C, control; vLPS, vehicle of LPS; vM, vehicle of mofezolac; M + vLPS, mofezolac and vehicle of LPS; vM + LPS, vehicle of mofezolac and LPS; M + LPS, mofezolac and LPS. ^§§§^*p* ≤ 0.001 compared with control or vLPS; ***p ≤ 0.001 compared with LPS alone.

Immunoblotting analysis was also performed to semiquantitatively evaluate both astrocyte and microglia activation in samples derived from mice groups previously described. In LPS-treated mice, a significant increase of GFAP expression was detected in all the brain regions tested when compared to controls or vehicle-LPS (Figure 4). Similar results were obtained for Iba-1 expression analysis (Figure 4).

Conversely, in mofezolac-treated animals previously injected with LPS, the GFAP as well as Iba-1 levels resulted significantly reduced in comparison to the animals that received LPS alone (Figure 4).

### Effect of the COX-1 Inhibitors—P6 and Mofezolac—on COXs Expression in BV2 Microglial Cell Line and Mice Brains

**Figure 5:**
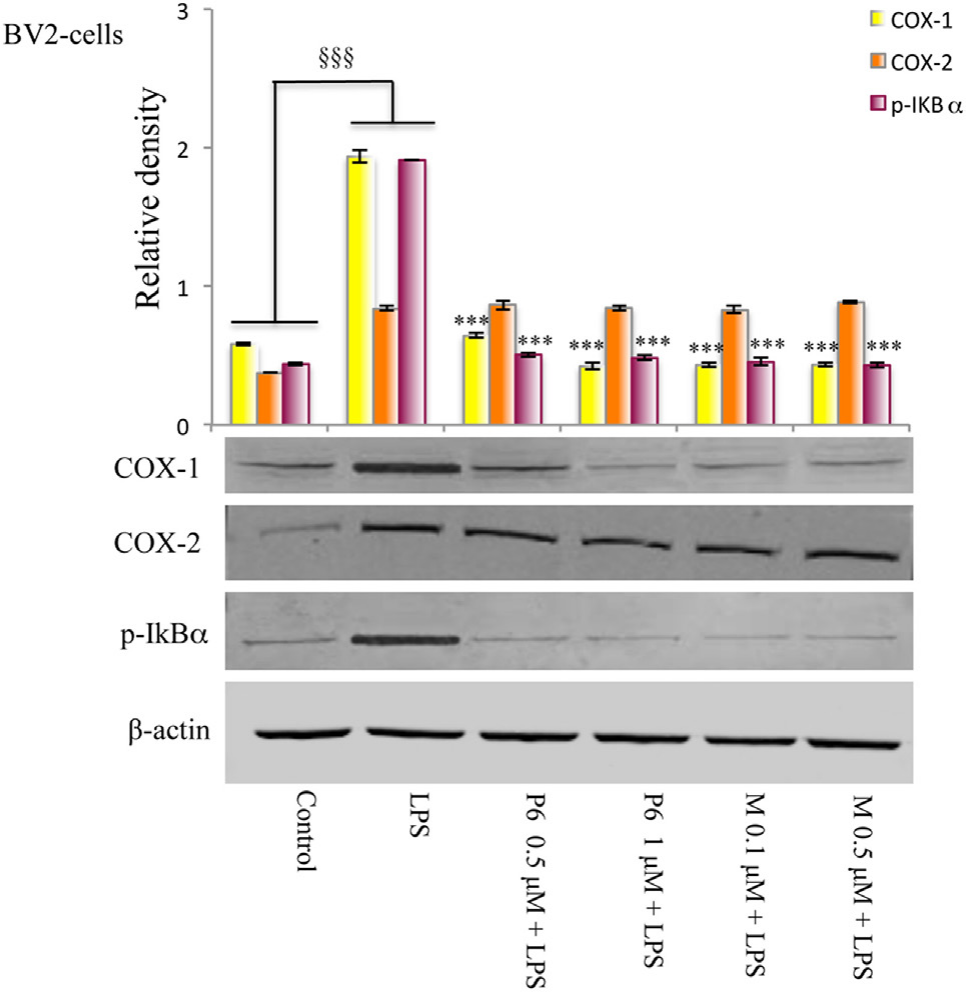
Effects of cyclooxygenase (COX)-1 inhibitors on the COXs expression and NF-kB phosphorylation induced by lipopolysaccharide (LPS) in BV2 microglial cells. Total protein was subjected to SDS-PAGE, followed by immunoblotting using pI*k*Bα antibody. The quantification of relative band intensities was expressed as relative density, after normalization against β-actin densitometry. Each bar represents the means ± SE of three independent experiments. BV2 microglial cells incubated with medium alone (control) or treated with LPS alone or in presence of the P6 and mofezolac (M) for 48 h. ^§§§^*p* ≤ 0.001 vs control, ***p ≤ 0.001 vs LPS alone.

The effect of mofezolac and P6 on COX-1 expression in LPS-treated microglial cells was evaluated by western blotting analysis. After 24 h, no significant difference between LPS-stimulated cells in the presence or absence of both COX-1 inhibitors was observed (data not shown). Interestingly, LPS-stimulated BV2 cells exhibited, at 48 h, increased levels of COX-1 expression in comparison to untreated cells (Figure 5). The COX-1 expression levels evaluated at 48 h of incubation with LPS was significantly reduced in 1 h pretreated cells with either selective COX-1 inhibitor P6 or mofezolac (Figure 5). Therefore, COX-1 expression was found to be time dependently reduced by both selective inhibitors tested.

The expression of COX-2 in the same cell lysates was also evaluated. Interestingly, COX-2 protein levels were unaffected by the presence of P6 or mofezolac. In these conditions, COX-2 expression resulted comparable to the level observed in cells stimulated with LPS alone (Figure 5).

Therefore, in LPS-treated BV2 microglial cell line, P6 and mofezolac were able to reduce COX-1 expression without affecting COX-2.

Cyclooxygenases expression was also *in vivo* evaluated, assaying different brain regions (Figure 4). LPS-challenged mice exhibited increased levels of COX-1 both in comparison to controls and vehicle of LPS (vLPS) administered animals in all the different areas tested (Figure 4). Interestingly, LPS-challenged mice that received mofezolac treatment exhibited a significantly reduction of COX-1 expression in all brain areas tested. Protein levels comparable to controls were detected in mice treated with vLPS + mofezolac, whereas in mice treated with the vehicle of mofezolac + LPS, COX-1 levels resulted comparable to those detected in LPS-treated mice (Figure 4).

Immunoblotting assay on tissues of the same brain areas was performed to test the *in vivo* effects of mofezolac administration on COX-2 expression extent. COX-2 resulted overexpressed in all tested brain regions in LPS-administrated mice. Interestingly, mofezolac exhibited no effect on COX-2 modulation in LPS-treated mice, being the protein levels almost comparable to LPS-treated mice (Figure 4).

### Effect of COX-1 Inhibitors on PGE_2_ Biosynthesis in BV2 Microglial Cells and Mouse Brain

The PGE_2_ biosynthesis extent was evaluated in supernatants of cell cultures at 48 h incubation time (Table 1A). PGE_2_ production in LPS-stimulated cells was significantly higher than its basal level present in the untreated cells (control). Interestingly, both COX-1 inhibitors were able to reduce in a dose-dependent manner PGE_2_ release in LPS-treated cells (Table 1A).

**Table 1:** *In vitro* and *in vivo* PGE_2_ release affected by cyclooxygenase (COX)-1 inhibitors P6 and mofezolac (M).

(A) Effect of COX-1 inhibitors P6 and mofezolac (M) on PGE_2_ release (ng/mL) at 48 h in lipopolysaccharide (LPS)-stimulated BV2 microglial cells					
**Control**	**LPS**	**0.5 µM P6 + LPS**	**1 µM P6 + LPS**	**0.1 µM M + LPS**	**0.5 µM M + LPS**

0.22 ± 0.025	0.97 ± 0.021^a^	0.21 ± 0.010^b^	0.12 ± 0.008^b^	0.06 ± 0.004^b^	0.03 ± 0.002^b^

**(B) Effect of mofezolac (M) on PGE_2_ release (ng/mg tissue) in specimens from LPS-treated mice**					

**Specimen**		**Control**	**Vehicle LPS**	**LPS**	**M + LPS**

Caudate–putamen		0.15 ± 0.050	0.73 ± 0.045^c^	5.61 ± 0.136^c^	1.84 ± 0.035^d^
Hippocampus		0.31 ± 0.036	0.69 ± 0.032^c^	4.55 ± 0.046^c^	2.33 ± 0.032^d^
Frontal lobe		0.33 ± 0.026	0.68 ± 0.026^c^	4.60 ± 0.055^c^	2.40 ± 0.046^d^
Substantia nigra		0.42 ± 0.021	0.74 ± 0.059^c^	5.48 ± 0.115^c^	3.31 ± 0.079^d^

*^a^p ≤ 0.01 compared with the control value*.

*^b^p ≤ 0.01 vs LPS alone*.

*^c^*p* ≤ 0.001 vs untreated animals (control)*.

*^d^p ≤ 0.01 vs LPS-treated animals*.

In the tested brain regions of LPS-treated mice, PGE_2_ levels were significantly increased with respect to control animals or mice receiving the vLPS (Table 1B). Interestingly, a significant reduction of PGE_2_ levels was detected in mofezolac-treated mice before LPS challenging (Table 1B).

### Effect of COX-1 Inhibitors on NF-kB Activation in LPS-Treated BV2 Microglial Cells and Mouse Brain

Since the phosphorylation and degradation of I*k*Bα, the inhibitory complex of NF-kB, is an essential step for NF-kB activation, the expression levels of the phosphorylated form of I*k*Bα (p-I*k*Bα) were evaluated. In this context, BV2 microglial cells exposed to LPS exhibited a significant increase of p-I*k*Bα in comparison with no stimulated cells (control) (Figure 5). Densitometric analysis revealed little p-I*k*Bα in the untreated cells, whereas pretreatment with all tested COX-1 inhibitors, significantly reduced I*k*Bα phosphorylation in LPS-stimulated cells (Figure 5).

Similar results were obtained in *in vivo* model. In fact, in all tested brain areas of LPS-treated mice, an increase of p-I*k*Bα, in comparison to control animals as well as to vLPS administered mice, was detected. Conversely, in mice receiving mofezolac (M), a significant reduction of p-I*k*Bα in all tested areas was detected (Figure 4).

p-I*k*Bα levels comparable to controls were detected in mice treated with vLPS + mofezolac, whereas in mice treated with vehicle of mofezolac + LPS, p-I*k*Bα levels were comparable to those detected in LPS-treated mice (Figure 4).

## Discussion

Clinical data and basic research outcomes showed a strict correlation between neurodegeneration and neuroinflammation (1, 8). COXs (both COX-1 and COX-2) play a central role in the inflammatory cascade by converting arachidonic acid (AA) into bioactive prostanoids (19). Both COX isoforms catalyze the same reactions: bis-oxygenation of AA to yield prostaglandin G_2_ (PGG_2_), and a peroxidase reaction, which converts PGG_2_ into prostaglandin H_2_ (PGH_2_). PGH_2_ is then transformed into PGE_2_, PGF_2α_, PGD_2_, PGI_2_, and thromboxane (TX) by specific terminal synthases. In the brain, both COX-1 and COX-2 are constitutively expressed (20), and there are regional differences in the regulation of COXs, probably as a consequence of the different distribution of COX-1 and COX-2 in the cerebral areas (21).

In physiological conditions, COX-1 is mainly expressed in microglia and perivascular cells, whereas COX-2 is found in postsynaptic dendrites and excitatory terminals, particularly in the cortex, hippocampus, and amygdala, with both neuronal and vascular associations. COX-1 recently has been recognized as a pivotal player in neuroinflammation (11, 22). Elevated levels of TXA_2_ and increased COX-1 expression in the rat hippocampus seem to be correlated to increased brain susceptibility to inflammation and neurodegenerative diseases (23). Moreover, in several models of neuroinflammation, COX-1 has been shown to support inflammatory processes facilitating pro-inflammatory PG upregulation, mainly PGE_2_ (22).

In this regard, we previously reported that the COX-1 selective inhibitor P6 was able to control the inflammatory response in LPS-treated N13 microglial cells, an *in vitro* model of neuroinflammation (14). In this context, P6 was able to selectively downregulate COX-1 protein expression, without affecting COX-2 levels, as well as reduce I*k*Bα phosphorylation. In this previous study, we proposed a new possible mechanism by which P6 negatively regulates COX-1 expression thereby NF-kB mediated signal-pathway modulation, suggesting the necessity of further studies to validate the efficacy of P6 as well as its analogs in the treatment of neuroinflammatory-based neurodegenerative disease (14).

Since the potential beneficial effect of COX-1 inhibition in the treatment of neuroinflammation has been considered, in the present study, we determined the modulatory effect of two selective COX-1 inhibitors—P6 and mofezolac—both in *in vitro* and *in vivo* models of neuroinflammation. For this purpose, we employed the mouse BV-2 microglial cells activated by LPS instead of N13 cell line to exclude a possible bias linked to the *in vitro* model used. Data of the present study clearly demonstrated that P6 was able to reduce COX-1 expression and negatively regulate the NF-kB activation, thus showing that these effects are independent from cell line used. Besides, P6 action on LPS-treated BV2 microglial cell responses was compared with the effects of mofezolac, an analgesic drug already approved to be used in humans, exerting its pharmacological action through a highly selective inhibition of COX-1 (24, 25). In this regard, mofezolac was able to reduce COX-1 expression in LPS-activated BV-2 cells accompanied to PGE_2_ release reduction and NF-kB activation downregulation.

More interestingly, these results were confirmed with those derived from the preclinical *in vivo* model of neuroinflammation, where mice intra-cerebroventricularly received LPS. Concerning *in vivo* investigation, GFAP and Iba-1 expression, two markers of neuroinflammation, were reduced by mofezolac to an extent depending on the encephalon area analyzed (see Results). Moreover, the increase of COX-1 expression observed in all the brain sections of LPS-treated mice was selectively downregulated by the *in vivo* treatment with mofezolac, as well as PGE_2_ release and I*k*Bα phosphorylation extent assayed in the tested brain areas.

In a previous work of Bosetti et al. carried out on a murine model, it was reported that evident neurodegeneration mediated by activated microglia and increased pro-inflammatory cytokines appeared in the hippocampus after LPS injection (11). Although, previous reports have shown sustained inflammation at 24 h after LPS injection (11, 26), in our *in vivo* model, we prolonged up to 72 h the time after LPS intraventricular injection to determine the optimal degree of inflammatory response. In fact, at 72 h after LPS injection, we clearly detected in all the brain regions tested, represented not only by hippocampus but also caudate, substantia nigra, and frontal lobe, the signs of neuroinflammation in terms of astrocytes and microglia activation.

Hippocampus is a part of the encephalon, located at the temporal lobe, where it plays a crucial role in processing information selected for the long-term memory; therefore, it is important to check what happens at level of this brain area the neuroinflammation modulation (27). Moreover, substantia nigra and caudate–putamen, both involved in neurodegenerative processes typically of the Parkinson’s disease, as well as frontal lobe, involved in superior cognitive pathways, were investigated to evaluate the effects of COX-1 inhibitors (28, 29).

Results from our experiments demonstrated that *in vivo* treatment with mofezolac was able to reduce glia activation acting selectively on COX-1 expression, thus supporting that COX-1 modulation represents a viable target useful to attenuate the neuroinflammatory response. In this regard, it was reported how COX-1 genetic depletion was able to attenuate microglial and astrocyte activation, reducing pro-inflammatory cytokine expression and preventing the neuronal cells loss in the hippocampus (11).

Recently, it was reported that the potent PET probe highly selective for COX-1, [^11^C]-radiolabeled ketoprofen methyl ester was able to detect activated microglia associated with amyloid plaque progression, suggesting the involvement of COX-1 in the neuroinflammatory process in AD (30). The role of COX-1 in neuroinflammation is also supported by the fact that COX-1 inhibition through drug or gene deletion reduces neuronal damage and inflammatory responses after injection of LPS or Aβ in mouse brain (31, 32). The LPS model is particularly relevant to examine activation of brain innate immunity, since it specifically and directly targets microglia, the immune resident cells in the brain, through the CD14 protein binding and subsequent toll-like receptor 4 mediated pro-inflammatory signaling pathway activation. These events cascade culminates to NF-kB activation leading to the release of cytokines, chemokines, ROS, PG, and TX (33, 34).

## Conclusion

Our results demonstrated that both P6 and mofezolac were able to reduce COX-1-derived PGE_2_ release complemented with an anti-inflammatory activity at the level of the NF-kB, thus providing mechanistic insights into the suppressive effect of these COX-1 inhibitors on LPS-induced neuroinflammatory response by microglia.

In conclusion, this work consolidated the hypothesis that selective COX-1 inhibitors can positively modify the inflammatory response in LPS-induced neuroinflammatory models. Overall these results, from *in vitro* and *in vivo* experiments, indicate the capability of two highly selective COX-1 inhibitors P6 and mofezolac to modulate the NF-kB signaling pathway. These findings emphasize the neuroprotective effect and therapeutic potential of COX-1 inhibitors useful in the control of neuroinflammatory diseases.

## Ethics Statement

This study was carried out in accordance with the recommendations of Ministero della Salute Decreto Ministeriale, Dott. Fabrizio Bertani. The protocol was approved by the Ministero della Salute Decreto Ministeriale no. 138/2014-B.

## Author Contributions

RC performed *in vitro* experiments with AC and RS; DL performed *in vivo* experiments with FN and LG; MGP designed the work and with PV prepared the tested compounds; GN supervised *in vivo* experiments; MAP supervised *in vitro* experiments and wrote the paper; and AS designed the work, supervised all the activities, and wrote the paper with help from other authors.

## Conflict of Interest Statement

The authors declare that the research was conducted in the absence of any commercial or financial relationships that could be construed as a potential conflict of interest.
